# The role of Aurora kinase A in hepatocellular carcinoma: Unveiling the intriguing functions of a key but still underexplored factor in liver cancer

**DOI:** 10.1111/cpr.13641

**Published:** 2024-04-08

**Authors:** Luca Grisetti, Clarissa J. C. Garcia, Anna A. Saponaro, Claudio Tiribelli, Devis Pascut

**Affiliations:** ^1^ Fondazione Italiana Fegato – ONLUS, Liver Cancer Unit Trieste Italy; ^2^ Department of Life Sciences Università degli Studi di Trieste Trieste Italy

## Abstract

Aurora Kinase A (AURKA) plays a central role as a serine/threonine kinase in regulating cell cycle progression and mitotic functions. Over the years, extensive research has revealed the multifaceted roles of AURKA in cancer development and progression. AURKA's dysregulation is frequently observed in various human cancers, including hepatocellular carcinoma (HCC). Its overexpression in HCC has been associated with aggressive phenotypes and poor clinical outcomes. This review comprehensively explores the molecular mechanisms underlying AURKA expression in HCC and its functional implications in cell migration, invasion, epithelial‐to‐mesenchymal transition, metastasis, stemness, and drug resistance. This work focuses on the clinical significance of AURKA as a diagnostic and prognostic biomarker for HCC. High levels of *AURKA* expression have been correlated with shorter overall and disease‐free survival in various cohorts, highlighting its potential utility as a sensitive prognostic indicator. Recent insights into AURKA's role in modulating the tumour microenvironment, particularly immune cell recruitment, may provide valuable information for personalized treatment strategies. AURKA's critical involvement in modulating cellular pathways and its overexpression in cancer makes it an attractive target for anticancer therapies. This review discusses the evidence about novel and selective AURKA inhibitors for more effective treatments for HCC.

## Graphical abstract

Aurora kinase A (AURKA) is a crucial protein that controls cell growth and division. It plays a major role in cancer development and progression, causing abnormal cell behaviour. AURKA is often overexpressed in hepatocellular carcinoma (HCC), making it a potential diagnostic and prognostic marker of disease severity. Increasing evidence is exploring the use of AURKA as a target for new anticancer treatments in HCC. Understanding the role of AURKA in cancer may improve liver tumour management and treatment, offering hope for better outcomes for patients in the future.

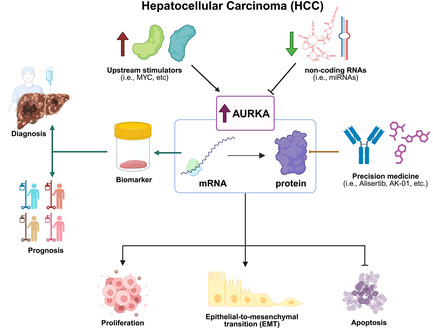

## 1 INTRODUCTION

In 2020, the World Health Organization (WHO) recorded about 905,677 liver cancer cases and approximately 830,180 deaths due to the disease (WHO, 2022). Hepatocellular carcinoma (HCC) is a primary liver tumour and represents approximately 75–85% of all liver malignancies. Furthermore, the incidence rate is expected to rise to over a million cases by 2025, posing a significant economic and public health burden.^1^, ^2^ HCC is a progressive disease with a dismal prognosis despite improvement in overall patient management, mainly developing within the context of chronic liver disease (CLD), notably cirrhosis.^3^ The majority of HCC patients are diagnosed with advanced tumour stages with a 5‐year survival of 20–40% and treatment options for HCC are limited relative to other types of solid tumours.^4^, ^5^

The underlying molecular mechanisms of HCC development remain elusive, further complicated by the inherent molecular and clinical heterogeneity observed in HCC, thereby adding another layer of complexity to fully elucidate the driving force triggering its development and progression. However, prominent genetic and epigenetic alterations play crucial roles in hepatocarcinogenesis.^6^ The surge in low‐cost sequencing technologies has paved the way for the identification of clinically relevant mutations resulting in altered signalling pathways. Among all, the alterations in cell cycle control machinery are heavily implicated in carcinogenesis. This complex machinery plays a central role in regulating when the cell should synthesize new DNA and proliferate or undergo growth arrest, DNA repair, or apoptosis.^7^, ^8^ Therefore, aberrantly expressed cell cycle proteins have become potential therapeutic targets in cancer, particularly following the FDA approval of cyclin‐dependent kinase (CDK)4/6‐specific inhibitors for HR+/Her2 – metastatic breast cancer.^9^, ^10^

Aurora kinases A, B, and C (AURKA, AURKB, and AURKC) are a family of serine/threonine kinases that function as critical regulators of mitotic cell division. Despite protein structure and kinase activity similarities, the three aurora kinases exhibit distinct cellular and subcellular localization. While all aurora kinases have been demonstrated to be amplified in many human malignancies due to their positive influence on mitosis, AURKA is unique in its well‐established role outside mitosis, which includes cell migration and invasion, epithelial‐to‐mesenchymal transition (EMT), DNA damage repair, and centrosome duplication.^11^, ^12^, ^13^, ^14^ Recent studies on AURKA's oncogenic role have laid the foundations for developing novel anti‐cancer therapies targeting this kinase, which may be more effective than conventional chemotherapeutic regimens.

## 2 AURKA IS A MASTER REGULATOR OF THE CELL CYCLE

AURKA plays a critical role in regulating the cell cycle and mitosis, which are essential in maintaining the integrity of genetic information.^15^ AURKA mutations have been associated with abnormal centrosome duplication and separation during mitosis, leading to defective spindle formation.^16^, ^17^

### 2.1 AURKA protein structure and domains

**FIGURE 1:**
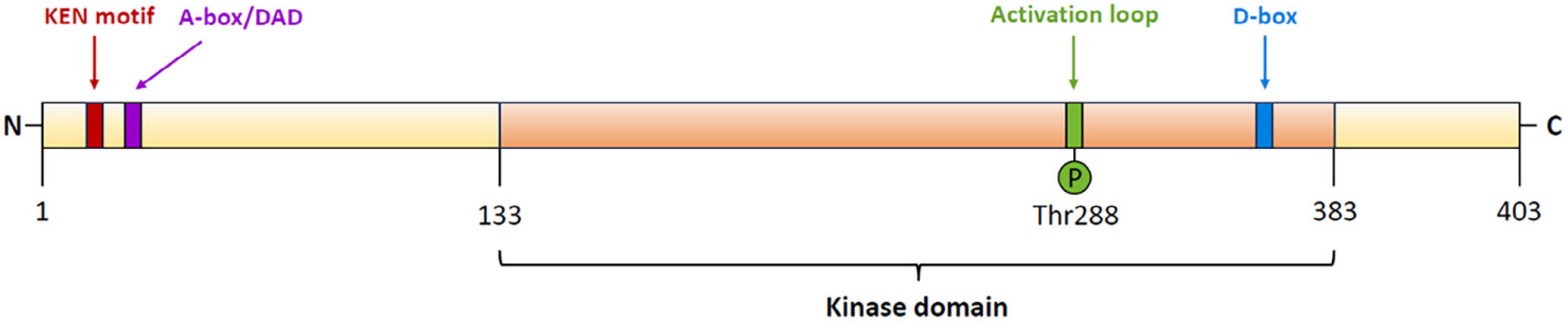
AURKA domains. AURKA is a protein composed of 403 amino acids (aa). The central region is the kinase domain (orange) flanked by two non‐catalytic domains (yellow), the N‐terminal domain and the C‐terminal domain. The green colour indicates the position of the conserved catalytic T‐loop residue (Thr288) required for AURKA activation. The N‐terminal domain and the C‐terminal domain contain two specific degradation boxes, the A‐box/D‐box activating domain (DAD) (purple) and the destruction box (D‐box) (blue), respectively. The N‐terminal domain contains the KEN box (red) binding site for CDH1‐APC/C required for AURKA degradation.

AURKA's gene maps to human chromosome 20q13.2 and encodes for at least 16 known transcript variants.^18^ The reference transcript encodes for a 403 amino acid (403aa) protein constituted by a kinase domain flanked by two non‐catalytic domains, the N‐terminal (length: 39‐139aa) and the C‐terminal (15–20aa) domains (Figure 1).^19^

**FIGURE 2:**
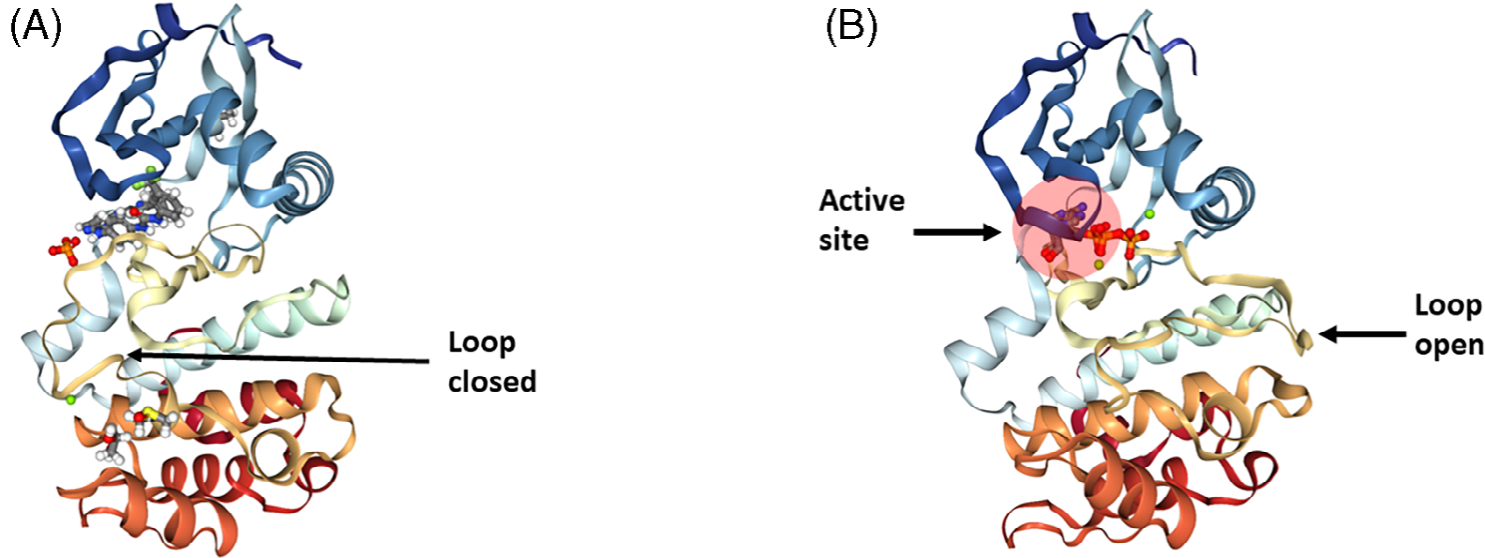
AURKA structure. (A) Human AURKA in a close conformation bound to the inhibitor CD532,^20^ structure 4j8M, taken from the protein data bank PDB database. (B) Human AURKA in an open conformation in complex with ATP,^21^ structure 5DNR, taken from the protein data bank PDB database. Phosphorylation on the activation loop switches the kinase into an active T‐loop conformation required to provide access to ATP and substrates.

The kinase domain is constituted by a β‐stranded N‐terminal lobe, an α‐helical C‐terminal lobe linked together by a hinge region, and an ATP‐binding pocket. The hydrogen bonds link the purine ring of adenosine to the hinge region.^19^ An intermolecular trans‐reaction within the two‐lobed AURKA domain induces autophosphorylation on the conserved catalytic T‐loop residue (Thr288) of the C‐terminal lobe, followed by a 3D conformation change of the kinase catalytic ATP‐binding pocket, thus opening the catalytic loop and activating the kinase activity (Figure 2).^22^, ^23^ The N‐terminal domain facilitates AURKA interaction with other proteins, thereby controlling AURKA localization and functions. The N‐terminal and the C‐terminal domains contain specific degradation boxes, A‐box/D‐box activating domain (DAD) and destruction box (D‐box), respectively (Figure 1), that mediate intra‐molecular interactions regulating AURKA degradation. They are recognized by the Cdc20 homologue 1 (Cdh1) protein, an activator of the Anaphase‐promoting complex/Cyclosome (APC/C).^24^, ^25^, ^26^, ^27^, ^28^, ^29^ The N‐terminal domain also contains the KEN motif (Figure 1), which is targeted by Cdh1‐APC and required for AURKA degradation.^29^

### 2.2 Regulation of AURKA transcription during the cell cycle

AURKA activity is prominent during the G2 phase of the cell cycle. Low levels of AURKA have been observed during the G1 phase, whereas an increase in transcription and protein accumulation in nuclei begins towards the end of the S phase and peaks during the G2 phase.^30^, ^31^

The transcription of AURKA is tightly regulated during the cell cycle through the cell cycle‐dependent element (CDE) and cell cycle gene homology region (CHR) localized in its promoter region.^30^ CHR localizes in the promoters of various late‐cell cycle genes interacting with the dimerization partner/retinoblastoma‐like/E2F/MuvB (DREAM) complex components. This complex regulates the genes involved in the cell cycle working either as a transcription activator or repressor, depending on the context. The binding of the DREAM complex to the CHR is facilitated by CDE, which is localized to four nucleotides upstream of the CHR.^32^ Besides AURKA, CDE/CHR elements are crucial for the transcription of several key regulators of the G2/M phase transition, including cyclin A, cell division cycle 25 (CDC25), CDK1, and polo‐like kinases 1 (PLK1).^33^, ^34^, ^35^ The coordinated action of CDE/CHR elements and the DREAM complex is fundamental for the precise transcriptional regulation of genes involved in the G2/M transition, thereby ensuring the proper execution of cell division.

AURKA can also be negatively regulated at the transcriptional level. Transcription factor SIX homeobox 3 (SIX3) and chromatin‐remodelling protein integrase interactor 1 (INI1/hSNF5) inhibit AURKA transcription by binding to specific sequences in *AURKA* promoter region.^36^, ^37^ Ribonuclease monocyte chemoattractant protein‐induced protein 1 (MCPIP1) can destabilize *AURKA* by cleaving the 3′ untranslated region (UTR).^38^

### 2.3 Regulation of AURKA activity during the cell cycle

**FIGURE 3:**
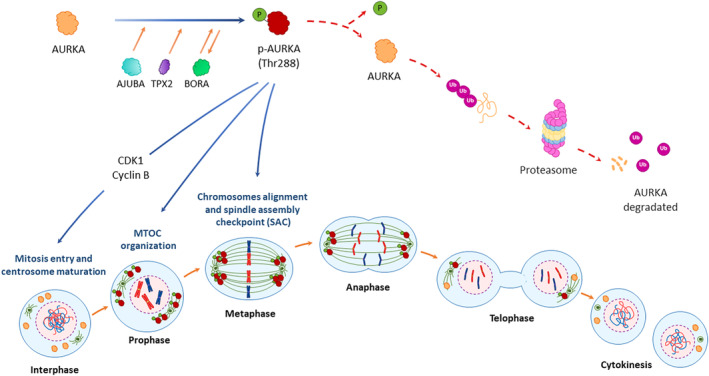
AURKA's roles during mitosis. AURKA is primarily activated by its auto‐phosphorylation on Thr288 [phospho‐AURKA (p‐AURKA)], which is promoted by several co‐factors including the Ajuba LIM protein (AJUBA), the Targeting protein for Xenopus kinesin‐like protein 2 (TPX2), and the Protein aurora borealis (BORA). Once activated, AURKA phosphorylates and activates Cyclin‐dependent kinase 1 (CDK1)‐Cyclin B complex to unlock the G2/M checkpoint. In the G2/M phase, AURKA can promote centrosome maturation, mitotic entry, mitotic spindle formation, spindle assembly checkpoint (SAC) establishment, and cytokinesis. As mitosis concludes, AURKA's degradation is mediated by APC/C through ubiquitin‐mediated proteolysis.

Numerous interactors can positively regulate AURKA levels throughout the cell cycle progression. During mitosis, AURKA autophosphorylation on Thr288 residue appears to prevail over any other form of regulation, although not always reflecting the active state of this kinase. This suggests that the autophosphorylation on Thr288 residue is necessary but insufficient for the total activation of AURKA, thus additional post‐translational modifications are required.^19^ Besides, AURKA is activated by the phosphorylation on Thr288 by several co‐factors including the ajuba LIM protein (AJUBA),^19^, ^39^, ^40^ TPX2 microtubule nucleation factor (TPX2),^19^, ^41^ and BORA aurora kinase A activator (BORA)^19^, ^42^ (Figure 3). AURKA can be phosphorylated on the same Thr288 residue by upstream kinases, such as the mechanistic target of rapamycin kinase (mTOR)^43^ or protein kinase A (PKA),^23^ possibly between interphase and metaphase. Nucleolar protein nucleophosmin can activate AURKA *via* Ser89 phosphorylation.^19^, ^44^ These phosphorylation events stabilize and safeguard AURKA against degradation throughout mitosis.^19^, ^45^

The phosphorylation on Thr288 triggers the activation of AURKA prompting the phosphorylation of its co‐factor, BORA, thereby increasing the kinase activity of AURKA.^42^ Consequently, AURKA can phosphorylate and activate the CDK1‐cyclin B complex to unlock the G2/M checkpoint.^39^ As the cell progresses into mitosis, the nuclear envelope disintegrates during the nuclear envelope breakdown, resulting in AURKA release from the nucleus and its predominant localization in centrosomes and proximal mitotic spindle (Figure 3). The N‐terminal domain facilitates the interaction of AURKA with the centrosomes in a microtubule (MT)‐dependent manner.^40^, ^46^ During prophase, the increase in CDK11 actively contributes to AURKA recruitment at the centrosomes,^47^ where it is crucial in organizing the MT‐organizing centre by recruiting and phosphorylating pericentriolar material proteins such as centrosomin, large tumour suppressor kinase 2 (LATS2), and BRCA1 DNA repair associated (BRCA1).^48^, ^49^, ^50^ LATS2 triggers the recruitment of γ‐tubulin and BRCA1 promotes MT nucleation during centrosome maturation.^48^, ^51^

During the initial spindle assembly, a positive feedback loop between AURKA and TPX2 confers AURKA its active conformation, protecting it against dephosphorylation by protein phosphatase 1 and protein phosphatase 6.^52^, ^53^, ^54^, ^55^, ^56^ TPX2 also favours the localization of AURKA in the proximity of the centrosome and spindle pole bodies.^45^ Crystallography studies showed that TPX2 induces a second conformational change on AURKA, generating a fully activated kinase able to interact with its substrates.^41^, ^52^, ^54^ Similar defects in spindle formation were observed in the case of TPX2 or AURKA depletion or inhibition,^57^, ^58^, ^59^, ^60^, ^61^ thus highlighting the prominent role of AURKA and TPX2 in both spindle formation and centrosome separation.^41^, ^52^

The degradation of AURKA and other cell cycle regulatory proteins typically occurs during “mitotic exit.” In this phase, AURKA is targeted for degradation by the ubiquitin‐proteasome system.^27^ Once activated, the APC/C, together with its co‐activator Cdh1, recognizes AURKA and facilitates the transfer of ubiquitin molecules to specific lysine residues^28^, ^62^ through the action of two E2 ubiquitin‐conjugating enzymes, ubiquitin‐conjugating enzyme E2 C (UBE2C) and UBE2S,^63^ promoting proteasome‐dependent protein degradation.^64^ The downregulation of AURKA activity and its ensuing degradation is essential to ensure correct cell cycle progression and prevent potential errors or anomalies.

## 3 AURKA DYSREGULATION IN HCC

The correlation between AURKA and malignant phenotypes has become clearer over time. According to the Genome Expression Profiling Interactive Analysis (GEPIA) database (http://gepia.cancer-pku.cn/), 82% of tumours (27 of 33 tumour types, including breast, uterus, ovaries, liver, and lung) show high levels of *AURKA* expression, with log2 (transcripts per million [TPM] +1) values ≥2. Conversely, only 18% of cancer types exhibit *AURKA* expression with log2 (TPM + 1) values <2 such as brain lower‐grade glioma, prostate adenocarcinoma, and thyroid carcinoma.^45^, ^65^

### 3.1 Expression pattern of AURKA in HCC

Numerous studies have investigated AURKA expression across publicly available gene expression datasets, all highlighting the higher expression of *AURKA* in tumours compared to non‐cancerous tissues (Table 1).^66^, ^67^, ^68^, ^69^, ^70^, ^71^, ^72^, ^73^, ^74^, ^75^, ^76^, ^77^, ^78^ *AURKA* showed a marked increase in Hepatitis B virus+ (HBV^+^) HCC samples compared to the paired HBV^+^ non‐tumoural liver tissues in GSE14520 [128.00 (78.79–219.79) vs. 26.35 (22.63–33.63)] and GSE121248 datasets [227.54 (155.42–335.46) vs. 80.45 (64.00–101.83)].^68^ By using integrated bioinformatics analyses, most of these studies have identified AURKA as one of the central hub genes with crucial functions in HCC.^68^, ^69^, ^70^, ^71^, ^72^, ^73^, ^75^, ^76^, ^77^ Other studies have independently supported this evidence, such as the research conducted by Jeng and colleagues. They observed *AURKA* overexpression in tumours compared to the distal portion of the liver collected from 137 HCC patients at the local hospital (Table 2).^79^

**TABLE 1:** List of gene expression datasets explored to assess *AURKA* expression.

AURKA expression	Dataset	HCC tissue	Control	Reference
Up	GSE14323	47 (HCV)	Adjacent:17 Cirrhosis: 41 Normal: 19	^66^
Up	GSE14520	225	Adjacent: 220	^66^, ^67^, ^68^, ^69^
Up	GSE25097	268	Adjacent: 243 Cirrhosis: 40 Normal: 6	^66^
Up	GSE45267	46	Adjacent: 41	^70^
Up	GSE101685	24	Normal: 8	^70^
Up	GSE84402	14	Adjacent: 14	^70^, ^71^
Up	TCGA	371	Normal: 50	^67^, ^69^, ^70^, ^72^, ^73^, ^74^, ^75^
	ICGC‐LIRI‐JP	138	Normal: 120	^75^
Up	GSE62232	81	Normal: 10	^70^, ^73^, ^75^, ^76^
Up	GSE121248	70 (HBV)	Adjacent: 37	^68^, ^71^, ^73^, ^77^
Up	GSE76427	115 HCC (46% with HBV, 54% with cirrhosis)	Adjacent: 52	^71^, ^73^
Up	GSE39791	72	Adjacent: 72	^73^
Up	GSE41804	20 (HCV)	Adjacent: 20	^73^, ^75^
Up	GSE54236	81	Adjacent: 80	^73^
Up	GSE57957	39	Adjacent: 39	^73^
Up	GSE64041	60	Adjacent: 60 Normal: 5	^73^
Up	GSE69715	8 Multiple specimens collected from the same patient (*n* = 45)	Adjacent:8 Multiple specimens collected from the same patient (*n* = 65)	^73^, ^75^
Up	GSE84005	38	Adjacent: 38	^73^
Up	GSE87630	64 (HBV)	Adjacent: 30	^73^
Up	GSE112790	185	Liver metastasis of colorectal cancer: 15	^73^, ^76^
Up	GSE89377	40	Normal: 13	^76^
Up	GSE47197	62 (HBV)	Adjacent: 62	^68^, ^77^
Up	GSE55092	31 (HBV)	Adjacent: 81	^77^
Up	GSE6764	35	Normal: 9 Dysplastic nodules: 17 Cirrhosis:14	^75^
Up	GSE107170	5 (HDV) 11 (HCV) 11 (HBV) Multiple specimens collected from the same patient (*n* = 118)	HDV cirrhosis: 7 Adjacent: 24 Multiple specimens collected from the same patient (*n* = 189)	^75^
Up	GSE12941	10	Adjacent: 10	^75^

**TABLE 2:** Literature evidence of the AURKA expression in HCC tissues.

Molecule	AURKA expression	HCC tissue	Control (Adjacent tissues)	References
mRNA	Up	10	10	^71^
mRNA	Up	244	199	^79^
mRNA	Up	46	46	^80^
Protein	Up			
mRNA and Protein	Up	3	3	^81^
Protein	Up	22	22	^82^
Protein	Up (WB)	24	24	^83^
	Up (IHC)	141	139	
mRNA	Up	40	40	^74^

Abbreviations: IHC, immunohistochemistry; WB, Western blot.

AURKA protein expression is often elevated within HCC tumours. Liu and colleagues noted an increase of AURKA in 24 tumour tissues compared to the matched adjacent tissues.^83^ Similarly, Shen and colleagues observed AURKA elevation in three HCC tissues (Table 2).^81^ Another study demonstrated that AURKA was significantly upregulated in 22 primary liver cancer tissues of HCC patients (77.3%, 17/22) (Table 2).^82^

Given its involvement in cancer, high levels of AURKA have been linked to clinical aggressiveness, poor outcomes, unfavourable prognoses, therapeutic resistance, and increased early recurrence in HCC patients.^80^, ^84^ The patients showing high AURKA expression (*n* = 52) had significantly shorter overall survival (OS) and disease‐free survival rates compared to the patients with low AURKA expression (*n* = 86) (Table 2).^83^ Chen and collaborators observed a poorer prognosis among patients with high *AURKA* levels (*n* = 15) compared to patients with low *AURKA* expression (*n* = 17). Approximately 40 months post‐surgery, ~45% of the patients with low AURKA levels survived compared to ~25% of patients with high AURKA levels (Table 2). They further underscored the importance of AURKA in HCC metastasis, noting that intrahepatic metastasis (*n* = 26) exhibited higher *AURKA* expression levels than primary HCC samples (*n* = 20) (Table 2).^80^

### 3.2 Expression pattern of AURKA in other liver diseases

Limited studies have been conducted on the expression of AURKA in pre‐tumoural conditions or benign liver diseases. In human samples, *AURKA* expression demonstrates a progressive increase from healthy individuals through CLDs to HCC cases.^85^ These observations are supported by findings from HBV‐transgenic mouse models developing HCC, where *Aurka* expression increases significantly during tumour development.^85^ In addition, AURKA expression was markedly increased in patients with liver fibrosis and a history of alcohol consumption compared to normal liver tissues.^86^

Multiple studies suggest that AURKA expression might play a role in hepatic steatosis and fibrosis. Overexpression of *aurka* in zebrafish promoted the expression of lipogenic factors and enzymes associated with steatosis, such as peroxisome proliferator‐activated receptor γ (*pparγ*), sterol regulatory element‐binding protein 1 (*srebp1*), and carbohydrate‐responsive element‐binding protein (*chrebp*).^87^ AURKA was increased in acetaldehyde‐stimulated hepatic stellate cells (HSC‐T6 and LX‐2 cells) and its inhibition by alisertib attenuated the high levels of actin alpha 2, smooth muscle, and collagen type I alpha 1 chain induced by acetaldehyde while increasing senescence‐associated beta‐galactosidase staining, a marker of cellular senescence. Thus, the authors hypothesized a potential positive role of AURKA in aggravating alcohol‐related liver fibrosis.^86^

AURKA has been implicated in enhancing HBV replication and expression in a kinase‐independent manner. AURKA knockdown inhibited viral replication and expression in HepG2 cells transfected with recombinant Adeno‐associated virus‐HBV (HBV1.3). A marked increase of viral DNA was observed in the kinase‐dead K162R mutant and the non‐phosphorylatable T288A mutant setups.^88^ Furthermore, a specific AURKA polymorphism (Ile31Phe) was associated with the susceptibility to HBV‐related HCC in the Chinese population.^89^

Despite limitations in available studies, these findings hint at the intriguing roles of AURKA in CLDs, which warrant thorough exploration to comprehensively understand the biological mechanism underlying their progression to liver tumours.

## 4 THE MOLECULAR MECHANISM OF AURKA DYSREGULATION IN HCC

Extensive evidence links *AURKA* overexpression to HCC, yet the precise molecular basis driving its involvement in hepatocarcinogenesis remains poorly understood. *AURKA* undergoes tight modulation both at transcriptional and post‐transcriptional levels.^90^, ^91^ Dysregulation of *AURKA* arises from various factors such as gene amplification, single‐point mutations, and non‐coding RNA modulations. This dysregulation culminates in the hyperactivation of AURKA, enabling its interaction with multiple proteins, including oncogenes and tumour suppressor genes, thereby fostering the development and progression of HCC.^92^, ^93^, ^94^, ^95^

**FIGURE 4:**
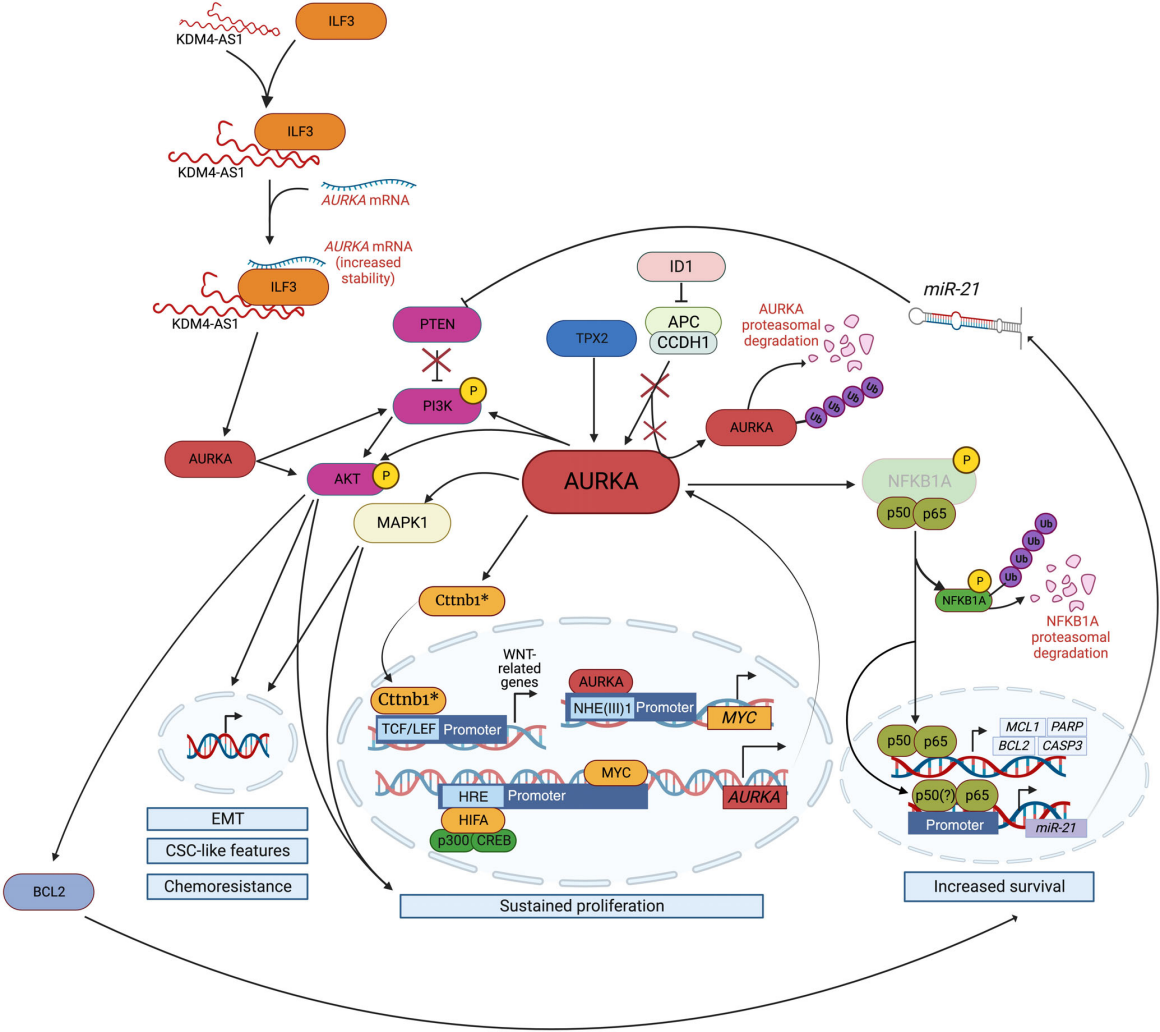
Summary of AURKA signalling network in hepatocellular carcinoma. AURKA plays a central role in regulating oncogenic pathways in hepatocarcinogenesis. AURKA transcription can be promoted by HIFA, under hypoxic conditions, and through a feedback loop involving MYC. The KDM4‐AS1/ILF3 complex stabilizes *AURKA*. Once the mature protein is produced, it is activated by TPX2 and shielded against degradation by ID1. AURKA can phosphorylate PI3K and AKT to promote gene expression involved in proliferation, EMT, CSC‐like characteristics, and drug resistance; while enhancing MAPK1 pathway activity. By phosphorylating NFKB1A, AURKA promotes its dissociation from the P50/p60 complex thus allowing its nuclear translocation and the transcription of pro‐survival genes (*MCL1*, *PARP*, *BCL2*, and *CASP3*). In addition, the complex mediates miR‐21 transcription. miR‐21 represses PTEN, thus contributing to sustaining PI3K/AKT signalling. AURKA positively regulates pro‐proliferative pathways by facilitating Ctnnb1 nuclear translocation, thereby promoting Wnt‐related gene transcription in Zebrafish models. Solid arrow, positive regulation; tapered arrow, translocation; inhibitor arrow, negative regulation; right angle arrow, transcription; transparent boxes indicate a negative effect.

*c‐Myc* is one of the most frequently upregulated genes in HCC, exerting a pivotal role in tumour initiation and progression.^96^ c‐Myc and AURKA mutually reinforce each other's expression at the transcriptional level in HCC. The mRNA expression levels of both genes exhibit a significant correlation in HCC clinical samples, including tumours versus paired non‐cancerous tissues, as well as in TP53‐mutated HCC specimens.^97^, ^98^ Mechanistically, MYC transcriptionally activates *AURKA* by binding to the highly conserved E‐box regions within the CpG islands of *AURKA*'s promoter. In vitro studies further suggest that MYC influences AURKA localization, promoting its nuclear accumulation over cytoplasmic distribution.^96^, ^98^ The MYC/AURKA signalling axis is exacerbated by the overexpression of the inhibitor of differentiation 1 (ID1), which competes with APC/C‐Cdh1 for binding, thereby impairing the ubiquitin‐mediated degradation of AURKA and facilitates HCC progression (Figure 4).^99^ Noteworthy, the MYC/AURKA interaction that often triggers cell transformation to a malignant phenotype is not exclusive to HCC. Various studies have demonstrated both the direct and indirect interaction between these oncogenes in several tumours including colorectal cancer, glioblastoma, triple‐negative breast cancer, and prostate cancer.^100^, ^101^, ^102^, ^103^ In a fibrolamellar carcinoma model, AURKA modulates the effects of upregulated PKA, consequently enhancing *MYC* expression.^104^ Additionally, the stability of *AURKA* is increased via the recruitment of interleukin enhancer binding factor 3 (ILF3) by the long‐non‐coding RNA, KDM4A antisense RNA 1 (KDM4A‐AS1), which is also elevated in HCC.^105^ Hypoxic conditions in HCC also trigger the hypoxia inducible factor 1 subunit alpha (HIF1A) to transcriptionally regulate *AURKA* by binding to the hypoxia‐responsive elements in the AURKA promoter and subsequently recruit the co‐activator p300/CREB binding protein.^106^ Forkhead box M1 (FOXM1), which along with AURKA is a co‐predictor of prognosis and sorafenib efficacy in HCC, regulates *AURKA* at the promoter level resulting in increased self‐renewal capacity of breast cancer stem cells.^74^, ^107^

*AURKA* variants have emerged as potential contributors to the initiation and progression of HCC across distinct populations. In a Taiwanese cohort study, individuals harbouring the *AURKA* rs1047972 (T/T) exhibited a 2.68‐fold higher risk of developing HCC than C/C homozygotes. The patients with A/T or A/A genotype at the rs2273535 were less susceptible to advancing to stage III/IV HCC, developing large tumours, and Child‐Pugh B or C grade, although they remained predisposed to liver cirrhosis. Additionally, carriers of the rs2064863 G/T + G/G genotype demonstrated a lower risk (*vs*. T/T carriers) of developing large tumours and Child‐Pugh grade B or C.^93^ Conversely, *AURKA* rs1047972 T/T (2.92‐fold) and T/C + T/T (3.38‐fold) genotypes were more prone to develop HCC compared to having the C/C genotype in an Egyptian cohort with Hepatitis C virus^+^ (HCV^+^) HCC.^94^

The dysregulation of specific non‐coding RNAs represents another mechanism of AURKA modulation. miR‐129‐3p is downregulated in HCC due to promoter hypermethylation, hinting at its tumour‐suppressive function in the liver.^108^, ^109^ miR‐129‐3p binds to *AURKA* 3'‐UTR, repressing its expression at the post‐transcriptional level. The diminished expression of miR‐129‐3p in HCC leads to aberrantly elevated *AURKA* levels.^109^ Similarly, other microRNAs including miR‐490‐3p, miR‐26a, and miR‐199b‐3p have been identified as repressors of *AURKA* by binding to its 3′‐UTR. In HCC, these miRNAs are downregulated thus unable to control AURKA's expression. The resulting upregulated AURKA expression positively influences various oncogenic processes including proliferation, migration, invasion (HepG2 and Hep3B),^110^, ^111^ and resistance to apoptosis (HepG2 and SK‐HEP1),^84^ beyond reducing the sensitivity of HCC cells (Huh7 and SMMC‐7721) to doxorubicin.^111^ In sorafenib‐resistant HCC cells, the negative modulator of AURKA, miR‐140‐5p, is sponged by the long non‐coding RNA metastasis associated lung adenocarcinoma transcript 1 (MALAT1), thus promoting the elevated expression of AURKA.^112^

## 5 AURKA REGULATES MULTIPLE CELLULAR PATHWAYS IN HCC

AURKA overexpression in HCC has been shown to profoundly influence various critical features of cancer cells, including proliferation, survival, migration, and invasion, indicating its significant involvement in disease development and progression. These cancer hallmarks are regulated by various signalling pathways – including but not limited to phosphatidylinositol‐3‐kinase (PI3K)/AKT and catenin 1 (CTNNB1) pathways – in which AURKA plays a critical role, leading to malignant transformation.^113^, ^114^, ^115^ As a cell cycle regulator, AURKA's role in promoting proliferation is not surprising, and its dysregulation can lead to aneuploidy and tumour development.^116^

### 5.1 AURKA regulates cell proliferation

Uncontrolled cellular proliferation is one of the key readouts associated with AURKA overexpression. Aside from its kinase activity, the nuclear localization of AURKA provides insights into its oncogenic role.

Chromatin immunoprecipitation data from HepG2 and BEL‐7402 revealed that AURKA can regulate MYC by binding to its nuclear hypersensitive element (NHEIII1) region, thus increasing its transcriptional activity in the nucleus (Figure 4). This indirect regulation was observed in a liver cancer cell line (MHCC‐97H) supporting the role of the ID1/AURKA/MYC axis in promoting a highly malignant phenotype with enhanced metastatic ability and drug resistance.^99^ Inhibiting either MYC or AURKA diminished the malignant phenotype of HCC cell lines^96^, ^99^ and the tumour growth in injected BALB/c nude mice models.^96^ Similar results were observed in a recent AURKA – small hairpin RNA knockdown study in HepG2 cells showing reduced proliferation and survival upon AURKA inhibition.^117^

Hypoxic conditions are crucial tumour microenvironmental factors that influence AURKA and HIF1A activity in HCC cells. In HepG2 and BEL‐7405 cells, AURKA promoted hyperproliferation, increased survival, and a more malignant phenotype by regulating downstream signalling AKT and p38/mitogen‐activated protein kinase (MAPK) pathways (Figure 4). AURKA silencing reduced the levels of both p‐AKT and p‐p38 proteins in treated cells thus attenuating all the downstream effects.^106^

In a zebrafish HCC model system, mutant‐induced *aurka* overexpression resulted in a reduction of membrane‐bound Ctnnb1, potentially signifying a higher transcriptional activity of Ctnnb1 in the mutant‐induced *aurka* (V352I) setup.^87^ Thus, sustaining the evidence of AURKA's role in Wnt/CTNNB1 signalling in the context of HCC.

### 5.2 AURKA mediates EMT and drug resistance

Upregulation of AURKA was observed in irradiated HCC cells, with concomitant overexpression of N‐cadherin and significantly elevated CD133 and CD44, characteristic features of EMT and cancer cell stemness (CSC), respectively.^80^ This bears clinical implications, particularly in HCC where a common phenomenon is the high refractoriness to standard chemotherapy, and even to targeted drugs.^118^

Similarly, by regulating PI3K/AKT and MAPK1 signalling pathways, AURKA determined an increase in cell invasiveness following irradiation in vitro (Figure 4). However, the latter study did not identify the specific protein directly regulated by AURKA.^80^, ^114^ Furthermore, the PI3K/AKT pathway mediates the effects of AURKA in promoting EMT in vitro (Hep3B and HuH7) and in vivo (BALB/C mice).^105^

### 5.3 AURKA promotes the resistance to apoptotic signalling

Resistance to apoptotic signalling is a common hallmark in HCC. The nuclear factor kappa B (NF‐κB) signalling is a prominently altered pathway in the development of HCC representing a master regulator of cell death and inflammation.^119^ AURKA promotes the classical activation of NF‐κB signalling by phosphorylating the NFKB inhibitor alpha (NFKBIA) on Ser32 and Ser36 residues, leading to the proteasomal degradation of the protein (Figure 4).^120^ This, in turn, allows the translocation of the NF‐κB complex into the nucleus and promotes transcription of pro‐survival target genes such as MCL1 apoptosis regulator BCL2 family member (MCL1), B‐cell lymphoma 2 (BCL2), poly(ADP‐ribose) polymerase (PARP), and caspase 3 (CASP3) (Figure 4). Previous studies have highlighted the unconventional role of CASP3 in promoting DNA damage, thus facilitating malignant transformation in MCF10A cells.^121^

The modulatory role of AURKA in the NF‐κB pathway further results in increased transcription of miR‐21, a non‐coding RNA that negatively regulates phosphatase and tensin homolog (PTEN) and blocks CASP3‐mediated apoptosis, through the upregulation of antiapoptotic proteins, such as p‐AKT and BCL2 (Figure 4). Zhang K. and colleagues first reported the potential clinical relevance of AURKA/NF‐κB/miR‐21/PTEN signalling axis wherein AURKA overexpression is a potential initiator of chemoresistance in vitro (HepG2, SMMC‐7721, and Hep3B) and in vivo mice models.^122^ Thus, this further adds another layer of complexity with AURKA's non‐canonical function extending towards the regulation of non‐coding RNAs.

## 6 TARGETING AURKA FOR LIVER CANCER THERAPY

Bioinformatics‐based approaches have identified AURKA as a key hub gene in HCC,^68^, ^69^, ^70^, ^71^, ^72^, ^73^, ^75^, ^76^, ^77^ rationalizing AURKA as a candidate therapeutic target. A recent study investigated the pharmacological mechanisms and internal factors influencing the efficacy of lenvatinib in HCC treatment. Lenvatinib is one of the first‐line oral multikinase inhibitors for advanced HCC, targeting vascular endothelial growth factor receptor 1‐3 (VEGFR1‐3), fibroblast growth factor receptor 1‐4 (FGFR1‐4), platelet‐derived growth factor receptor alpha (PDGFRA), KIT proto‐oncogene, receptor tyrosine kinase (KIT), and rearranged during transfection (RET).^123^, ^124^ In the study, docking algorithms were used to predict the non‐covalent interaction between molecular targets and the drug identifying AURKA as one of the possible targets of lenvatinib in HCC.^123^

Over the years, various studies identified several compounds able to inhibit AURKA in vitro, with some accumulating sufficient evidence to undergo further evaluation as potential cancer therapies during preclinical or clinical trials. The initial generation of AURKA inhibitors comprises ATP‐competitive inhibitors that bind to the ATP binding pocket of AURKA, and currently represent the majority of AURKA inhibitors that have undergone clinical investigation.^125^ Newer types of AURKA inhibitors bind to an allosteric site, impeding either the kinase activity or AURKA protein–protein interactions.

Pan‐aurora kinase inhibitors have also been developed to target multiple Aurora family members (AURKA, AURKB, and AURKC) with moderate specificity. More than 10 Pan‐aurora kinase inhibitors have recently been designed and tested in vitro and in vivo in preclinical studies particularly in solid tumours, such as AKI‐001, SCH‐1473759, and BPR1K871, exhibiting potent inhibitory effects on aurora kinase activity with IC50 values below 50 nM.^126^, ^127^, ^128^ Although these molecules have been tested in relevant models, results in clinical studies are yet to confirm these drugs as AURKA inhibitors with *bona fide* clinical utility.

Alisertib (MLN8237), an ATP‐competitive and reversible AURKA‐specific inhibitor, is the most extensively studied AURKA inhibitor. It exhibits higher selectivity (200‐fold higher) for AURKA than for AURKB. Cell‐based phenotypic studies revealed that alisertib promotes cell arrest at the G2/M phase, disorganized mitotic spindle, and chromosome misalignment.^129^, ^130^ In multiple cancer cell lines and xenograft models, alisertib inhibits cell proliferation by impairing mitosis, blocking the EMT process, inducing cell cycle arrest and autophagy, as well as accelerating cancer cell apoptosis and senescence.^129^, ^131^ Due to its potent efficacy in preclinical studies, alisertib was tested in phase I/II clinical trials for several cancers, including lymphomas, leukaemia, gastric, ovarian, and breast tumours. However, phase III trials were halted in 2015 due to the lack of significant effects on patient survival and the presence of strong side effects including hematologic‐ and gastrointestinal‐related toxicities, commonly observed in single‐agent alisertib studies.^132^, ^133^ Hence, alisertib has been considered for combination therapies to enhance its anti‐tumour efficacy and reduce its toxicity with manageable adverse effects. A phase I clinical trial demonstrated that a combination of alisertib and docetaxel was well tolerated and exhibited antitumor activity in various cancer types, such as prostate cancer and upper gastrointestinal adenocarcinomas.^134^, ^135^ Similarly, promising results were observed with the combination of alisertib and paclitaxel in ovarian cancer, hinting that a lower dose of alisertib could yield better outcomes.^136^

In pre‐clinical studies conducted in HCC models, alisertib has been tested in combination with lenvatinib.^137^, ^138^ Alisertib increased the cytotoxic effects and anti‐metastatic activity of lenvatinib in Hep3B (p53‐deletion) and HepG2 (wild type), which may suggest that this combinatorial treatment may be effective regardless of p53 mutational status.^82^ Similarly, the simultaneous inhibition of AURKA and heat shock transcription factor 1 (HSF1) with danusertib and KRIBB11, respectively, resulted in increased apoptosis of HCC cells (HepG2 and Bel‐7402) presumably through the activation of the endoplasmic reticulum stress response. Consequently, co‐administration of danusertib and KRIBB11 in xenografted nude mice showed a reduction in Ki‐67 expression, signifying constrained proliferation of HCC cells, and a slow tumour growth rate.^81^

Although alisertib is the most extensively studied AURKA inhibitor, other AURKA‐selective inhibitors have entered the market. AK‐01 (LY3295668), which first entered clinical trials in 2014, is a reversible ATP‐competitive inhibitor harbouring a fluorine atom within the pyrimidine ring which aids in orienting carboxylate of AK‐01 closer to Thr217 of AURKA, thus facilitating its inhibition. In preclinical models, Du and colleagues demonstrated that AK‐01 exhibits promising efficacy, specifically by blocking proliferation in a time‐dependent and affecting the cell viability in a broad range of cancers, including HCC‐derived cell lines.^139^ These observations were confirmed in an in vitro model of HCC where the treatment with AK‐01 reduced cell viability and determined a cell cycle arrest coupled with some defects in cytokines.^140^ AK‐01 is currently undergoing phase II clinical trials (NCT03092934) in patients with small‐cell lung cancer, breast cancer, and other solid tumours^139^ (https://clinicaltrials.gov).

## 7 THE POTENTIAL ROLE OF AURKA AS A BIOMARKER IN HCC

Despite technological advancements, concerted preventive campaigns, and surveillance initiatives in recent decades, only a restricted number of patients receive an early‐stage diagnosis. Most HCC patients are diagnosed with advanced tumour stages, yielding a 5‐year survival rate of only 20–40%.^141^ Moreover, in the last three decades, numerous systems have been developed to stratify HCC patients and classify them into prognostic groups. The Barcelona Clinic Liver Cancer (BCLC) staging system relies only on tumour characteristics (e.g., number and dimensions of the nodules), preserved liver function, and clinical variables (such as total bilirubin, portal hypertension, and presence of ascites), excluding the use of biomarkers.^142^ On the contrary, the Cancer of the Liver Italian Program^143^ and the Groupe d'Etude et de Traitement du Carcinome Hépatocellulaire staging from France^144^ included alpha‐fetoprotein (AFP) as a biomarker. The BALAD score considers bilirubin, albumin (ALB), lens culinaris agglutinin A–reactive fraction of AFP (AFP‐L3), AFP, and des‐gamma‐carboxy‐prothrombin (DCP).^145^ However, the significant heterogeneity in HCC underscores the limitations of the available systems in comprehensively stratifying the patients. Based on these premises, there remains a crucial clinical need for identifying and integrating novel diagnostic and prognostic biomarkers for HCC.

### 7.1 AURKA as a tissue biomarker associated with HCC

Multiple studies showed a marked upregulation of *AURKA* in HCC compared to paired distal tissues or healthy livers (Table 1),^66^, ^67^, ^68^, ^69^, ^70^, ^71^, ^72^, ^73^, ^74^, ^75^, ^76^, ^77^, ^78^, ^79^, ^80^, ^81^, ^82^, ^83^ suggesting the diagnostic utility of *AURKA* in HCC.

The analysis of the GSE121248 dataset that collected gene expression data of 70 HBV^+^ HCC tumour samples and 37 adjacent normal tissues revealed that *AURKA* could discriminate HCC nodules from the normal adjacent tissues with an area under the curve (AUC) value of 0.897 (Table 3).^78^ The investigation of five public datasets (GSE6764, GSE41804, GSE62232, GSE107170, and TCGA datasets) identified differentially expressed genes associated with HCV^+^ HCC. *AURKA* was able to distinguish HCV^+^ HCC patients from healthy controls in three datasets (GSE69715, GSE107170, and TCGA‐LIHC), with AUCs higher than 0.71 (TCGA‐LIHC dataset, AUC = 0.986),^75^ thus demonstrating the consistency of *AURKA* as a potential diagnostic biomarker.

**TABLE 3:** The diagnostic and prognostic value of AURKA in HCC and its correlation with immune infiltration.

Biomarkers	Datasets	Risk score model	Significance of the marker	Immune correlation
**AURKA as a diagnostic biomarker**				
*AURKA* ^78^	GSE121248 (HCC *n* = 70; adjacent normal tissues *n* = 30) (HBV^+^ HCC)		AUC = 0.897.	
*AURKA* ^75^	GSE69715 (*n* = 103), GSE107170 (*n* = 75), and TCGA‐LIHC (*n* = 79) (HCV^+^ HCC)		GSE69715: AUC = 0.719 (95%CI: 0.604–0.834). GSE107170: AUC = 0.850 (95%CI: 0.765–0.935). TCGA‐LIHC: AUC = 0.986 (95%CI: 0.968–1.000).	
**AURKA as a prognostic biomarker**				
*AURKA* ^79^	National Taiwan University Hospital (1983–1997) (*n* = 224)		*AURKA* overexpression is associated with grades II–IV and stages IIIB–IV disease.	
*AURKA* ^66^, ^67^, ^78^, ^146^, ^147^	TCGA (*n* = 362)		OS: HR = 1.9, cut‐off = median, *p* = 0.00028 (*n* = 181 vs. *n* = 181).	
*AURKA* ^66^, ^70^, ^78^, ^147^	TCGA (*n* = 362)		DFS: HR = 1.6, cut‐off = median, *p* = 0.001 (*n* = 181 vs. *n* = 181).	
*AURKA* ^73^, ^76^, ^146^, ^148^	TCGA (*n* = 364)		OS: HR = 1.77 (1.25–2.50), cut‐off = best performing, *p* = 0.0011; Low‐*AURKA* (*n* = 220) vs. High*‐AURKA* (*n* = 144).	
*AURKA* ^75^	ICG‐LIRI‐JP (*n* = 112)		OS: HR = 1.95 (1.39–2.75), *p* = 0.00233.	
*AURKA* ^77^	GSE47197, GSE55092, and GSE121248 (HBV^+^ HCC) (*n* = 111)		OS: HR = 2.79 (1.30–6.02), *p* = 0.0061.	
*AURKA*, *TPX2*, *CDK1*, *PLK1*, *DLGAP5*, *CDC20*, *BIRC5*, *TACC3*, *CENPA* ^66^	TCGA (*n* = 340) and ICG‐LIRI‐JP (*n* = 243)		1‐, 2‐, 3‐year OS: TCGA‐training set (*n* = 240): AUC = 0.81 (95%CI: 0.73–0.88) at 1 year; AUC = 0.69 (95%CI: 0.61–0.78) at 3 years; AUC = 0.66 (95%CI: 0.56–0.77) at 5 years. TCGA‐validation set (*n* = 100): AUC = 0.81 (95%CI: 0.69–0.93); AUC = 0.71 (95%CI: 0.57–0.86); AUC = 0.93 (95%CI: 0.85–1.00). ICG‐LIRI‐JP dataset: AUC = 0.65 (95%CI: 0.48–0.82); AUC = 0.69 (95%CI: 0.58–0.80); AUC = 0.70 (95%CI: 0.59–0.81).	CD4 + T cells, macrophages, neutrophils, and dendritic cells. Immune checkpoints: *SIGLEC15*, *TIGIT*, *CD274*, *HAVCR2*, *PDCD1LG2*.
*AURKA*, *SPC25*, *MCM2*, *NUF2*, *BLM* ^72^	TCGA (*n* = 369)	(0.3497 * *SPC25*) + (0.0995 * *MCM2*) + (0.0327 * *NUF2*) + (0.0369 *AURKA) + (0.3185 * *BLM*)	OS: AUC: 0.89 (*n* = 373) 5‐year OS: Training cohort (*n* = 239): high‐risk group = 38% vs. low‐risk group = 71%, *p* = 0.00024. Validation cohort (*n* = 130): high‐risk group = 32% vs. low‐risk group = 40%, *p* = 0.01.	
*AURKA*, *PZP*, *RACGAP1*, *ACOT12*, *LCAT* ^149^	TCGA (*n* = 365), ICGC (*n* = 227), and a clinical cohort (*n* = 59)	(0.0996 * *AURKA*) − (0.1421 * *PZP*) + (0.3809 * *RACGAP1*) − (0.0742 * *ACOT12*) − (0.1438 * *LCAT*)	1‐, 2‐, 3‐year OS TCGA dataset: AUC = 0.741 at 1 year; AUC = 0.724 at 2 years; AUC = 0.718 at 3 years. ICGC dataset: AUC = 0.727; AUC = 0.720; AUC = 0.725. Clinical cohort: AUC = 0.803; AUC = 0.707; AUC = 0.701.	CD4^+^ T cells and macrophages.
*AURKA*, *CCNB1*, *NEK2*, *RACGAP1* ^75^	ICGC‐LIRI‐JP (*n* = 123) (HCV^+^ HCC)	(0.6819 * *CCNB1*) + (0.8859 * *NEK2*) − (1.3715 * *RACGAP1*) + (0.4831 * *AURKA*)	3‐year OS: AUC = 0.778.	
*AURKA*, *FOXM1* ^74^	TCGA (*n* = 341) and KMUH (*n* = 30)		5‐year OS (TCGA): high‐expression group (*FOXM1* ^*high*^ + *AURKA* ^*high*^) = 23%, opposite‐expression group (*FOXM1* ^*high*^ + *AURKA* ^*low*^/*FOXM1* ^*low*^ + *AURKA* ^*high*^) = 50%, low‐expression group (*FOXM1* ^*low*^ + *AURKA* ^*low*^) = 47%, log‐rank *p* < 0.001. 5‐year OS (KMUH): high‐expression group = 22%, opposite‐expression group = 76%, low‐expression group = 79%, log‐rank *p* = 0.004. 5‐year DFS: high‐expression group = 22%, opposite‐expression group = 29%, low‐expression group = 32%, log‐rank *p* < 0.001.	
			5‐year OS (Sorafenib‐treated TCGA, *n* = 29): high‐expression group = 0%, opposite‐expression group = 28%, low‐expression group = 67%, log‐rank *p* = 0.042.	
*AURKA* ^146^	TCGA (*n* = 370)			B cells (*r* = 0.451, *p* = 1.06e‐18), CD4^+^ T cells (*r* = 0.608, *p* = 2.77e‐36), CD8^+^ cells (*r* = 0.305, *p* = 7.37e‐ 09), macrophages (*r* = 0.262, *p* = 7.71e‐07), neutrophils (*r* = 0.096, *p* = 7.58e‐02), and dendritic cells (*r* = 0.262, *p* = 7.8e‐07).
*AURKA* ^147^	TCGA (*n* = 362)			B cells (*r* = 0.451, *p* < 0.0001), CD4+ T cells (*r* = 0.157, *p* < 0.01), macrophages (*r* = 0.262, *p* < 0.0001), and dendritic cells (*r* = 0.466, *p* < 0.0001).
*AURKA*, macrophage expression level^147^			OS: HR (*AURKA* ^high^ + low macrophage level vs. *AURKA* ^high^ + high macrophage levels) = 1.64, *p* = 0.0283. After removing confounding factors, OS: HR = 2.08, *p* = 0.0061.	

Abbreviations: ACOT12, acyl‐CoA thioesterase 12; BLM, BLM recQ like helicase; CCNB1, cyclin B1; LCAT, lecithin‐cholesterol acyltransferase; MCM2, minichromosome maintenance complex component 2; NEK2, NIMA‐related kinase 2; NUF2, NUF2 component of NDC80 kinetochore complex; PZP, PZP alpha‐2‐macroglobulin like; RACGAP1, Rac GTPase activating protein 1; SPC25, SPC25 component of NDC80 kinetochore complex.

### 7.2 The role of AURKA as a prognostic biomarker in HCC

Jeng and colleagues observed that *AURKA* overexpression in HCC patients was associated with high‐grade disease (grades II–IV) and portal vein tumour invasion (stages IIIB–IV),^79^ representing one of the first studies to suggest a possible role for *AURKA* as a prognostic biomarker for HCC patients. By querying the TCGA database, other studies showed that patients with higher *AURKA* expression had shorter OS [Hazard ratio (HR) = 1.9, *p* < 0.001]^66^, ^67^, ^78^, ^146^, ^147^ and DFS (HR = 1.6, *p* = 0.001)^66^, ^70^, ^78^, ^147^ compared to low‐*AURKA* group (*n* = 181 vs. *n* = 181) using the median *AURKA* expression as the cut‐off value (Table 3). A slight improvement in HR was obtained when the best‐performing cut‐off value was selected to divide the population into two groups. The survival analysis demonstrated that patients with low *AURKA* levels (*n* = 220) had longer OS [HR = 1.77 (1.25–2.50), *p* = 0.001], compared to the ones with high *AURKA* (*n* = 144) (Table 3).^73^, ^76^, ^146^, ^148^

The analysis of *AURKA* expression, in correlation with the patient survival in the ICGC‐LIRI‐JP cohort (HCV^+^ HCC samples, *n* = 112), revealed that patients with higher *AURKA* expression had shorter OS [HR = 1.95 (1.39–2.75), *p* = 0.002] (Table 3).^75^ Similarly, the higher levels of *AURKA* in the nodules of HBV^+^ tumours were associated with shorter OS [HR = 2.79 (1.30–6.02), *p* = 0.006, *n* = 111] (Table 3).^77^ All these observations reinforce the potential value of this kinase as a prognostic marker for HCC.

### 7.3 The potential of gene signatures to predict HCC patients' prognosis

Recent evidence included *AURKA* among gene signatures able to stratify patients based on their expected survival time.^66^, ^72^, ^74^, ^75^, ^149^ AURKA and eight related genes [*TPX2*, *CDK1*, *PLK1*, DLG‐associated protein 5 (*DLGAP5*), *CDC20*, baculoviral IAP repeat containing 5 (*BIRC5*), transforming acidic coiled‐coil containing protein 3 (*TACC3*), and centromere protein A (*CENPA*)] were markedly upregulated in HCC and associated with shorter OS and DFS in the TCGA dataset.^66^ The risk model based on the nine‐gene signature showed a potential value in predicting the prognosis of HCC in three different cohorts (TCGA‐training set, TCGA‐validation set, and ICG‐LIRI‐JP dataset) with AUCs higher than 0.65, 0.69, and 0.66 at 1, 3 and 5 years, respectively (Table 3).^66^ Similarly, other studies obtained an improvement in the stratification and the prognosis prediction of HCC patients when including multiple genes in their risk models (Table 3).^72^, ^75^, ^149^

The coordinated expression of *AURKA* and *FOXM1* was associated with patient prognosis in the TCGA dataset (*n* = 341). The 5‐year OS was less than half in patients with high expression of the two genes compared to patients with low expression (Table 3). Apart from its role in HCC prognosis, the potential of *AURKA* in combination with *FOXM1* was evaluated in sorafenib‐treated patients (*n* = 29). Patients with high *AURKA* and *FOXM1* expression have a shorter OS (5‐year OS: 0%), while the low‐expression patients have a longer survival (5‐year OS: 67%). Thus, *AURKA* and *FOXM1* expression was associated with patient prognosis and could represent a predictor of sorafenib efficacy.^74^

### 7.4 The immune infiltration reinforces the prognostic role of AURKA

AURKA can have a role in immune cell recruitment and immune microenvironment modulation, which determines the immune response to the tumour, thus possibly influencing prognosis.^66^, ^146^, ^147^, ^149^ The signature composed of *AURKA*, PZP alpha‐2‐macroglobulin like (*PZP*) Rac GTPase activating protein 1 (*RACGAP1*), acyl‐CoA thioesterase 12 (*ACOT12*), and lecithin‐cholesterol acyltransferase (*LCAT*) was able to determine the prognostic risk of the patients. Interestingly, the low‐risk score group exhibited a reduced macrophage M0 and an increased proportion of resting memory CD4^+^ T cells within tumours (Table 3).^149^

Utilizing the TIMER database (https://cistrome.shinyapps.io/timer/), *AURKA* expression was positively correlated with the infiltration of several immune cells in the HCC microenvironment, including B cells, CD4^+^ T cells, macrophages, and dendritic cells (Table 3).^146^, ^147^ Islam and colleagues also suggested a positive correlation with CD8^+^ T cells and neutrophils.^146^ Moreover, the higher expression of the gene signature comprised by *AURKA* and the 8‐related genes was associated with a higher percentage of immune cell infiltration (CD4^+^ T cells, macrophages, neutrophils, and dendritic cells) (Table 3) and higher expression of the immune checkpoints [sialic acid binding Ig like lectin 15 (*SIGLEC15*), T cell immunoreceptor with Ig and ITIM domains (*TIGIT*), programmed death ligand 1 (*CD274*), hepatitis A virus cellular receptor 2 (*HAVCR2*), and Programmed cell death 1 ligand 2 (*PDCD1LG2*)] (Table 3).^66^ Interestingly, the combination of *AURKA* expression and macrophage levels showed a promising prognostic value for HCC patients (TCGA dataset, *n* = 362) (Table 3). A high macrophage level predicted a markedly shorter OS (HR = 1.64, *p* = 0.028) in the high‐*AURKA* group (Table 3); and adjusting for confounding factors such as age, stage, gender, race, and tumour purity, the impact of macrophage level on OS in the high‐*AURKA* group became stronger (HR = 2.08, *p* = 0.006) (Table 3).^147^

Whether considered as a single factor or as a part of a gene signature, AURKA can be associated with immune infiltration.^66^, ^146^, ^147^, ^149^ This association provides valuable insights into the prognostic potential of AURKA in HCC, thus possibly improving personalized treatment strategies among HCC patients.

## 8 SERUM AURKA AS A BIOMARKER FOR CANCER

To our knowledge, studies on the potential utility as a biomarker of serum AURKA expression in HCC and other tumour types remain lacking. No significant differences in AURKA levels were observed between breast cancer patients and controls. However, upon investigating the relationship between AURKA levels and clinical‐pathological features, significant differences emerged based on lymph node status (N0 vs. N1 + N2).^150^ Analysis of serum AURKA levels using ELISA kits in 119 women with breast cancer prior to neoadjuvant treatment demonstrated predictive value in the treatment response. Elevated pre‐treatment serum AURKA levels were significantly associated with a more favourable response to neoadjuvant treatment (*p* = 0.039). Moreover, multivariate analyses revealed that serum AURKA level ≥4.75 ng/ml was correlated with a higher complete pathological response rate (OR: 3.5; 95% CI: 1.2–10.1; *p* = 0.023).^151^

Similarly, AURKA levels were evaluated in 92 patients with nasopharyngeal carcinoma (NPC) and 93 healthy individuals. NPC patients displayed significantly higher AURKA levels compared to healthy controls (0.8283 ± 0.0089 vs. 0.8189 ± 0.0098, *p* < 0.001). Integrating these findings with other parameters, a nomogram model was developed to predict NPC risk. The nomogram model exhibited high predictive accuracy, with an AUC of 0.897 (95%CI: 0.848–0.947) and 0.770 (95%CI: 0.628–0.912) in the training and validation set, respectively.^152^

Serum AURKA shows promise as a biomarker in various tumours, thus additional studies are warranted to assess its potential clinical utility in HCC.

## 9 CONCLUSIONS

AURKA is a serine/threonine kinase that plays a pivotal role in mitosis. It has become gradually evident that AURKA's functions extend beyond regulating the cell cycle in cancer. Exploiting these cancer‐related pathways can offer a promising avenue for anti‐cancer therapies, especially with the identification of several AURKA inhibitors.

The initial studies with AURKA inhibitors have paved the way for novel therapeutic strategies, particularly in combination with other drugs targeting different hallmarks of HCC. AURKA's potential role in modulating immune checkpoints and influencing immune cell infiltration within the HCC microenvironment suggests that combining AURKA inhibitors with immune checkpoint inhibitors could be an effective therapeutic approach for patients.

## AUTHOR CONTRIBUTIONS

Conceptualization: DP. Literature search: LG, CJCG, and AAS. Writing – original draft preparation: LG, CJCG, and AAS. Writing – review and editing, DP, CT. Supervision: DP. Funding acquisition: DP. All authors have read and agreed to the published version of the manuscript.

## FUNDING INFORMATION

LG was supported by a Ph.D. scholarship from the University of Trieste. DP is supported by the EU grant C3B, Programma di cooperazione Interreg V‐A Italia‐Slovenia 2014–2020. CJCG is supported by a scholarship from the Department of Science and Technology (DOST) – PCHRD, Bicutan, Taguig City. AAS and CT are supported by an intramural grant from the Italian Liver Foundation.

## CONFLICT OF INTEREST STATEMENT

The authors declare no conflict of interest.
